# Botulinum Neurotoxin Therapy for Depression: Therapeutic Mechanisms and Future Perspective

**DOI:** 10.3389/fpsyt.2021.584416

**Published:** 2021-04-23

**Authors:** Yang Li, Tong Liu, Weifeng Luo

**Affiliations:** ^1^Department of Neurology, The Second Affiliated Hospital of Soochow University, Suzhou, China; ^2^Institute of Pain Medicine and Special Environmental Medicine, Nantong University, Nantong, China; ^3^College of Life Sciences, Yanan University, Yanan, China

**Keywords:** depression, botulinum neurotoxin, hippocampus, brain-derived neurotrophic factor, 5-HT

## Abstract

Depression is one of the most common mental disorders, which causes global burden. Antidepressants and psychotherapies are the mainstay of treatment for depression, which have limited efficacy. Thus, alternative approaches for preventing and treating depression are urgently required. Recent clinical trials and preclinical researches have clarified that peripheral facial injection of botulinum neurotoxin type A (BoNT/A) is a rapid, effective and relative safe therapy for improving some symptoms of depression. Despite its safety and efficacy, the underlying therapeutic mechanisms of BoNT/A for depression remains largely unclear. In the present review, we updated and summarized the clinical and preclinical evidence supporting BoNT/A therapy for the treatment of depression. We further discussed the potential mechanisms underlying therapeutic effects of BoNT/A on depression. Notably, we recently identified that the anti-depressant effects of BoNT/A associated with up-regulation of 5-HT levels and brain-derived neurotrophic factor (BDNF) expression in the hippocampus in a preclinical mouse model. In summary, these studies suggest that BoNT/A therapy is a potential effective and safe intervention for the management of depression. However, fundamental questions remain regarding the future prospects of BoNT/A therapy, including safety, efficacy, dose-response relationships, identification of potential predictors of response, and the precise mechanisms underlying BoNT/A therapy.

## Introduction

Major depressive disorder (MDD) is a complex mental disease, which is characterized by symptoms of emotional, motivational, cognitional, and physiological domains (1). MDD is a highly prevalent disease of mental disorders which ranging from 6 to 18% across different countries, and it has substantially increased since 1990, possibly driven by global population growth and aging (2). According to World Health Organization (WHO), it was estimated that more than 350 million people suffer from MDD all over the world (3). In addition, many studies indicate women have been shown to be at greater risk for MDD than men (4). MDD is one of the top ten causes of disability all over the world. And MDD has been predicted to be a leading cause of global disease burden by 2030, in view of the overall disability and sufferings caused by it (5). For instance, the economic costs due to depression worldwide is estimated to be 1.15 trillion US dollars per year (3), and it is estimated that in the United States alone, this figure exceeds US$210 billion, resulting in 45% direct costs, 5% costs related to suicides and 50% costs related to work (3).

The symptoms of depression include low mood, decreased interest in the daily activities, decreased motivation, appetite and sleep disturbance, psychomotor agitation or retardation, cognitive impairment, and suicidal thought (6, 7). In addition, the patients with MDD have poorer physical health, including increased prevalence of cardiovascular disease, diabetes, and premature mortality compared with the general population (8). MDD which was untreated or partially treated has prodigious influence for the patients, their family, health-care system, and society (9, 10).

Clinically, the current treatments for depression are pharmacological and psychological interventions. Early clinical observations indicated that decreased monoamine function in the brain contributed to the pathogenesis of depression. Thus, antidepressants were developed in order to up-regulate monoamines levels in the brain either by inhibiting neuronal reuptake of them or by inhibiting their degradation. Although antidepressants are typically more efficacious than placebo in many clinical trials, some evidence suggested that ~50% of patients with depression were not responsive to antidepressant treatments (11). In addition, antidepressant medication may cause significant side effects, such as weight gain, increased risk of diabetes, and sexual dysfunction. Notably, cognitive behavioral therapy is also shown to have only moderate therapeutic effect on depression (12). Given the enormous disease burden of MDD and limited efficacy of current antidepressants or cognitive behavioral therapy, there is an imminent need to develop an alternative effective therapy for depression. To this end, there is growing evidence supporting botulinum neurotoxin type A (BoNT/A) therapy as useful method to treat major depression (13–16). In the present review, we have provided clinical and preclinical evidence supporting BoNT/A therapy for treatment of depression and discussed the potential therapeutic mechanisms and future perspectives.

### Overview of Botulinum Neurotoxins

Botulinum neurotoxins (BoNTs) are produced by *Clostridium botulinum*, of which there are 7 identified and different serotypes (A-G) (17, 18). BoNTs has a molecular weight of 150 kDa, which consist a light chain (LC; 50 kDa) and a heavy chain (HC; 100 kDa) (19–21). The main action of BoNTs occurs in the neuromuscular junction. In botulism poisoning, flaccid paralysis occurs by inhibiting the release of neurotransmitters from the peripheral cholinergic nerve terminals of the skeletal and autonomic nervous system (20). BoNTs are a typical example of bacterial exotoxins which target intracellular substrates. BoNTs have developed a structural organization aiming at delivering the metalloprotease domain into the host cell cytosol and by exploiting several physiologic functions of nerve terminals can achieve it (21). When local injection of BoNTs, they have limited diffusion, and their action can be reversible with time. Based on this above-mentioned feature, BoNTs (especial BoNT/A) have become the safe and most efficacious treatment for various kinds human syndromes that are characterized by hyperactivity of nerve terminals (21).

The classical mechanisms underlying the actions of BoNT/A is the inhibition of acetylcholine (ACh) release from the presynaptic nerve terminals, then reducing the activity of muscle fibers (22, 23). There are five major steps of BoNT/A transport in the nerve terminal, including binding and specificity, internalization into nerve terminals, membrane translocation, inter-chain disulfide reduction and SNARE protein cleavage (20), especially endocytosis, intracellular trafficking and transcytosis are important. It has been reported that HC/A can bind to and activate fibroblast growth factor receptor 3 (FGFR3), which is a tyrosine kinase receptor, in neuroblastoma cells (24). Recently, it was reported that by using non-toxic full-length BoNT/A(0) mutant, a catalytically inactive, BoNT/A(0) enters cortical neurons via a pathway dependent on FGFR3 receptor (25). Further study showed that BoNT/A(0) enters neurons through both dynamin-dependent and dynamin-independent endocytosis (25).

BoNT/A may also act similarly to the action of tetanus neurotoxins on the nervous system, which is the well-known example of retro-axonal transport inside motor axons (26). It is noteworthy that peripheral injection of BoNT/A can have a transynaptic action on the central neuronal circuits, including the spinal cord and higher brain regions (27, 28). So far, convincing evidence supporting BoNT/A1 retrograde transport to the CNS was supplied by tracking down the cleavage of the SNARE within the CNS neurons after peripheral injection, using a specified antibody for the novel epitope generated from the cleavage of SNAP-25 by BoNT/A1 (17, 29, 30). By injecting BoNT/A1 into the rat whisker pad, it caused the appearance of truncated SNAP-25 in the somatodendritic area of primary efferent facial motoneurons (31). It was also found that after the catalytically active BoNT/A was injected into the nasolabial muscle tissue of rats and mice, it was transported to the facial nucleus (FN) in the brain (27). Retrograde transportation of BoNT/A1 can also occur through primary sensory afferents, as it was observed that arrival of truncated SNAP-25 is not only in the trigeminal nucleus (29) but also in the spinal cord dorsal horn after subcutaneous or intramuscular injection (30). The transportation of BoNT/A1 includes undergoing retrograde from periphery to the ganglia and anterograde from ganglia to the afferent innervations in the brain stem and/or the spinal cord (28). Therefore, these results suggested that the intracellular transportation of BoNT/A1 may be an active retro-axonal transport, rather than through their passive diffusion or diffusion of split productions (31).

### Clinical Application of BoNTs in Neurology

**Figure 1:**
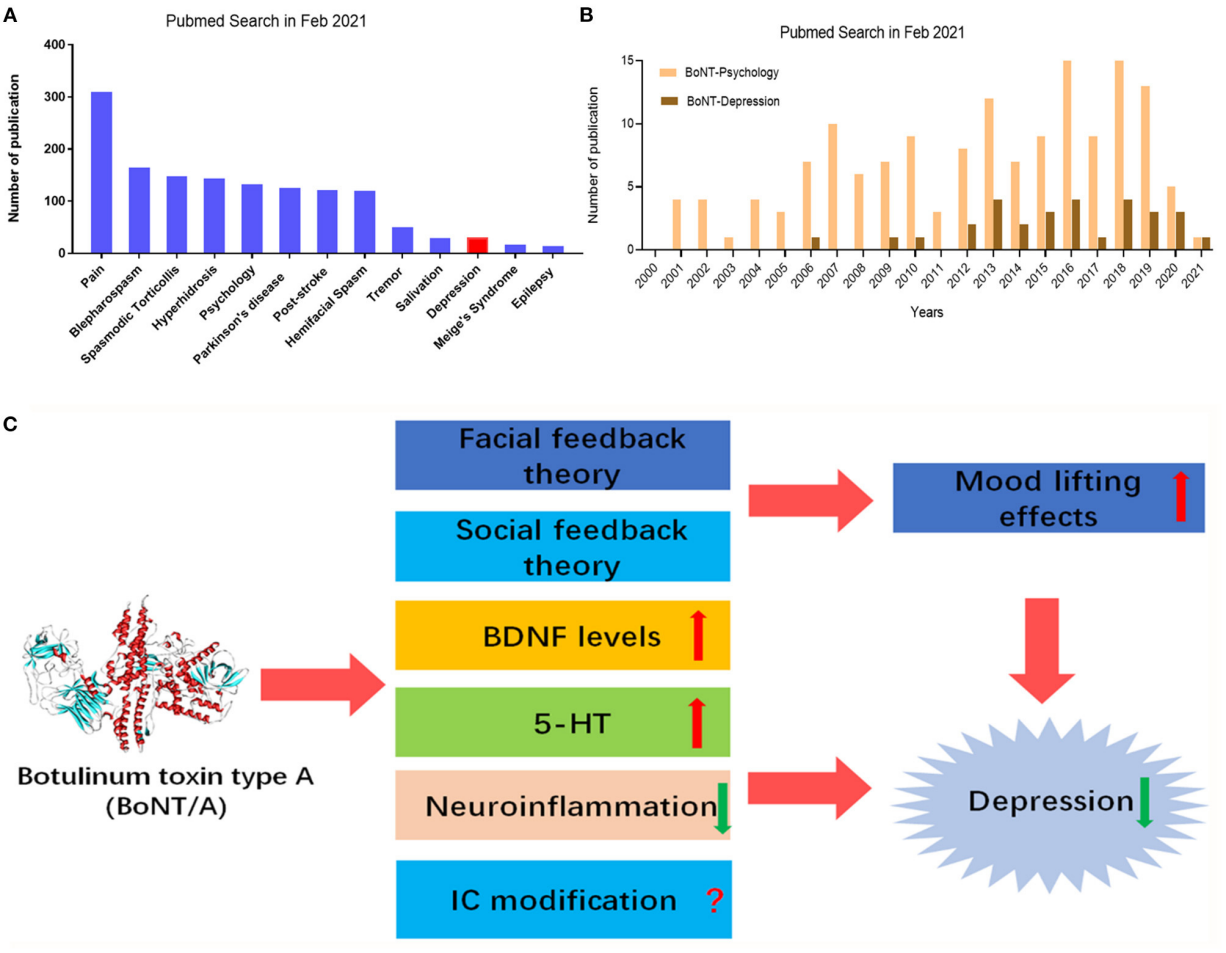
PubMed search for BoNT/A and neurological diseases, including depression. **(A)** Number of publications applying BoNT/A in Parkinson's disease, pain, hyperhidrosis, post-stroke, tremor, salivation, epilepsy, spasmodic torticollis, hemifacial spasm, blepharospasm, Meige's Syndrome, depression, and psychology. **(B)** Number of publications for applying BoNT/A in depression and psychology in the last 20 years. **(C)** Therapeutic mechanisms of BoNT/A in depression. Peripheral injection of BoNT/A produces antidepressant effects possible though multiple mechanisms, such as facial feedback and/or social feedback to lift mood, IC modifications, up-regulation of BDNF levels, increasing 5-HT levels, and dampening neuroinflammation. IC, insular cortex; BDNF, brain-derived neurotrophic factor; 5-HT, 5-hydroxytryptamine.

Currently, BoNTs therapy is widely administrated in clinical neurology, including dystonia, spasticity, autonomic disorders, and chronic pain (22, 23, 26) (Figure 1A). BoNTs can not only block the skeletal neuromuscular transmission, but also the autonomic innervation (24). Therefore, BoNTs therapy may also be beneficial for hyper-hidrotic disorders, urologic or gastrointestinal disorders (21). BoNT/A therapy had been applied for the management of many neurological disorders, such as dystonia and spasticity (25). Recently, a meta-analysis study including a total of 42 clinical trials reassessed the efficacy of BoNTs for movement disorders treatment, such as blepharospasm, hemifacial spasm, and laryngeal dystonia (27, 28). BoNT/A therapy was though as a choice of the pharmacological treatment in focal spasticity to improve limb position, functional ability and to release pain (29, 30). The application of BoNTs within the broad category of autonomic indications includes the hypersecretory disorders, such as hyperhidrosis and sialorrhea (31). Preclinical and clinical evidence have showed that BoNTs therapy had analgesic effects on neuropathic pain, trigeminal neuralgia, and chronic migraine (32). Notably, BoNT/A has been given official approval for preventive therapy of chronic migraine by Food and Drug Administration (FDA) in pain medicine (33). BoNTs can alleviate trigeminal neuralgia and can last for 6 months or more (34). Recently, BoNTs is also applied for the management of motor and non-motor symptoms in Parkinson's disease (PD) patients (35). Intriguingly, recent meta-analysis and literatures support that clinical application of BoNT/A may have antidepressant properties (14, 36, 37) (Figure 1B). In the current review, we summarized the clinical and preclinical studies on BoNT/A-induced antidepressant effects, discussed the therapeutic mechanisms, and proposed the future directions of BoNT/A therapy for depression.

### BoNT/A Therapy for Depression: Clinical Evidence

Although pharmacological and psychological treatment for depression are available, a considerable proportion of depressed patients are resistant to current standard treatment (38, 39). In esthetic medicine, the injection of BoNT/A in the glabellar region is a commonly used intervention (40). The contraction of the corrugator muscles is able to produce glabellar frown lines, which is also required for the facial expression of negative emotion, such as anger, fear, and/or sadness (41). Thus, it was concluded that BoNT/A therapy can actually make facial expression of emotion less negative, suggesting BoNT/A therapy may have mood-lifting effects in some depressed patients.

In 2006, Finzi et al. first reported open-label application of BoNT/A in 10 depressed patients led to significant improvement of self-rated depression score by using Beck Depression Inventory II (BDI-II) (15). Two months after the injection of BoNT/A, nine patients were not depressed and one patient reversed negative mood. Although there is no control experiment and the number of cases is too small, this study inspired people to further investigate the therapeutic effects of BoNT/A on depression. Subsequently, there are several randomized controlled trials (RCT) of BoNT/A therapy for the management of depression. In addition, injection of BoNT/A into frown muscles and glabellar region in humans was performed in most studies. Other injection sites were also increasingly used, such as bilateral lateral canthus (42) in humans. The injection sites of five reports were glabellar regions and the concentration of the injection were 29 U applying in females and 39 to 40 U for males. After that, Montgomery Asberg Depression Rating Scale (MADRS), BDI-II, and Hamilton Depression Scale (HAMD) were employed for the clinical evaluation of depression. They found that patients had a remission rate of more than 50% following BoNTs treatment, while females had a higher rate of remission than males (13). However, this study lasted only 6 weeks. In 2018, Finzi's another study showed that BoNTs injection was also effective for bipolar depression in men (43).

A randomized and placebo-controlled trial was conducted to evaluate the possible beneficial effect of injecting BoNT/A into the glabellar area as an adjuvant therapy for antidepression (44). This study lasted 16 weeks, making up for the shortcomings of abovementioned Finzi's research. Thirty participants randomly joined into the BoNT/A-treated (*n* = 15) or saline-treated (*n* = 15) group. The response rates of BoNT/A and placebo groups were 60.0 and 13.3%, respectively. This trial suggested that a single injection of BoNT/A in the glabellar region may rapidly reach an intense and sustained remission for some depressed patients, who did not response to former medication. This study is relatively perfect, except for a small number of samples. Lewis et al. assessed the effect of BoNT/A on mood by comparing the patients who received BoNT/A therapy in glabellar region and who had other cosmetic treatment (45). Total 25 female participants participated in this study. BoNT/A treatment group was lower than the control group in the scores of Irritability-Depression-Anxiety Scale (IDAS). For some participants, their first BoNT/A treatment was 2 weeks prior to the measurement, however one participant first BoNT/A treatment was 6 years ago. In order to avoid large skewness, the geometric mean is used to measure the central trend. The geometric mean was 195 days, which is the longest remission duration.

Brin et al. evaluated the antidepression effect of BoNT/A using two doses (30U and 50U) in females (36). At week 6, BoNT/A (50 U) treatment group did not separate from placebo group for any parameters. BoNT/A (30 U), administered in a standardized injection pattern in a single session, had a consistent efficacy signal across multiple depression symptom scales for 12 weeks or more. BoNT/A (30 U)/placebo MADRS differences of (observed ANCOVA) was ≥4.0 points (up to week 15) and ≥2.0 points (weeks 18–24), which is agree with the 2-point change threshold considered clinically relevant in MDD.

Magid et al. evaluated the effect of BoNT/A lasting 24 weeks (46). The response rates were 55% (6/11) in the BoNT/A-first group, 24% (4/17) in the BoNT/A-second group, and 0% (0/19) in the placebo group, respectively. The results suggested that the first injection of BoNT/A significantly decreased the BDI scores compared to that before treatment. An additional dose (no more than 20 U) were secondly injected to those patients who still had a severity score or greater for glabellar frown lines (47). Together, clinical studies (summarized in Table 1) have demonstrated that BoNT/A may be effective for the management of depression.

**Table 1:** Clinical trials and preclinical studies on botulinum toxin in the treatment of depression.

**Year**	**Authors**	**Periodicals**	**study**	**Sex**	**Number**	**Groups**	**Brands**	**Doses**	**Dissolution**	**Injection**	**Tests**	**Results**	**Duration**
2006	Finzi et al. (15)	Dermatol Surg	RCT	female	10	BoNT/A	—	29 U	—	Frown muscles	—	Nine of ten experienced resolution of their depression symptoms.	2 months
2009	Lewis et al. (45)	J Cosmet Dermatol	RCT	female	25	Placebo-BoNT/A	—	—	—	Frown muscles	IDAS	BoNT/A-treated group increased by 6 points.	195 days
2012	Wollmer et al. (44)	J Psychiatr Res	RCT	female and male	30	Placebo-BoNT/A	Botox Cosmetic, Allergan	29U/female, 39U/male	0.9% NaCl solution, 100U/2.5 ml	Glabellar region	HAMD, BDI	Response rate 60.0 vs. 13.3%	16 weeks
2013	Hexsel et al. (47)	Dermatol Surg	RCT	—	25	Depressed–non-depressed	Botox, Allergan Inc, Irvine, CA	20U	0.9% NaCl solution, 100U/1 ml	Glabellar region	BDI	BDI scores were significantly lower than before.	12 weeks
2013	Finzi et al. (13)	J Psychiatr Res	RCT	female and male	74	Double-Blind,placebo-BoNT/A	Botox Cosmetic, Allergan	29U/female, 39U/male	0.9% NaCl solution, 100U/1 ml	Frown muscles	MADRS	Response rates at 6 weeks from the date of injection were 52% and 15% in the OBA and placebo groups.	6 weeks
2014	Magid et al. (46)	J Clin Psychiatry	RCT	female and male	30	Double-Blind, placebo-BoNT/A	—	29U/female, 39U/male	0.9% NaCl solution, 40U/1 ml	Glabellar region	BDI, HDRS-21	Response rates were 55% (6/11) in the BTA-first group, 24% (4/17) in the BTA-second group, and 0% (0/19) in the placebo group.	24 weeks
2018	Chugh et al. (43)	J Psychiatr Pract	RCT	female and male	37	Chronic and treatment resistant depression	—	29U/female, 39U/male	—	Glabellar region	BDI, HDRS-21, MADRS	Almost all of the patients (41/42) showed clinically meaningful improvement in the symptoms of depression.	3 weeks
2018	Finzi et al. (48)	J Psychiatr Res	RCT	female and male	6	Bipolar depression	—	29–46 U	—	Glabellar region	BDI	This is the first report of successful BT therapy of bipolar depression in six patients.	15 weeks
2019	Li et al. (14)	Neurosci Bull	Animal testing	male	5–10 per groups	Placebo-BoNT/A- fluoxetine	BoNT/A, Lanzhou, China	0.18 U	0.9% NaCl solution, 0.18U/100 μl	mouse cheeks	TST, FST	BoNT/A improves depressive-like behaviors in mice undergoing spatial restraint stress.	2 weeks
2020	Brin et al. (36)	Int Clin Psycho!!breakpharmacol	RCT	female	255	double-blind placebo-controlled	—	30 U50U	0.9% NaCl solution	—	MADRSH!!breakAMD-17	Neither BoNT/A 30 U nor 50 U demonstrated statistically significant superiority over placebo at the primary endpoint, but 30 U showed consistent.	24 weeks

*BDI, Back depression inventory; BoNT/A, botulinum neurotoxin type A; FST, forced swim test; HAMD, hamilton depression scale; IDAS, irritability-depression-anxiety scale; MADRS, Montgomery Asberg depression rating scale; RCT, randomized controlled trial; TST, tail suspension test*.

### BoNT/A Therapy for Depression: Preclinical Evidence

To date, preclinical animal studies of BoNTs treatment for depression are still limited, but there are some clues. A single facial injection of BoNT/A induced a rapid and continuous improvement about depression-like behaviors in naive and space-restriction-stressed (SRS) mice. The antidepressant-like effects of BoNT/A was reflected by a decreased duration of forced swimming test and tail suspension test immobility (14). Techniques currently used to assess antidepressants effects by using rodent models include olfactory bulbectomy, chronic mild stress, chronic forced swim test, novelty-suppressed feeding, novelty-induced hypophagia, social defeat stress, and learned helplessness (49). Given that different models have different sensitivity to behaviors tests, using different animal models to validate the anti-depressant effects of BoNT/A may be necessary in the future.

To date, the clinical trials and animal studies of BoNTs treatment of depression are still relatively lacking, and the number of cases needs to be increased to evaluate its safety and efficacy, especially the long-term efficacy. However, it was noticed that the publication about the application of BoNTs in depression is increasing (Figure 1B). Subsequently, we will discuss the therapeutic mechanisms underlying the effects of BoNTs on depression.

### Therapeutic Mechanisms of BoNT/A in Depression

To date, the potentially mechanisms underlying the therapeutic effects of BoNT/A in the treatment of depression is still elusive. There are some review article or meta-analysis that discussed its efficacy and possible mechanisms (37, 50). Based on current clinical and preclinical evidence, several hypotheses were proposed to explain the therapeutic mechanisms of BoNT/A on depression to date. The facial feedback hypothesis claims that the injection of BoNT/A between the eyebrows interfere with emotional feedback. Because botulinum toxin can paralyze muscles, it is impossible to modify facial expression according to mood states, such as happy, sadness and anger, which often appear in depressed patients. Social feedback hypothesis indicated that happy facial expressions will get positive social feedback and improve mood. Finally, facial injection of BoNT/A causes structure or function changes in the brain to alleviate depression, for example, upregulation of brain-derived neurotrophic factor (BDNF) expression in the brain (51). We will further discuss these abovementioned hypotheses as follow (Figure 1C).

### Facial Feedback Hypothesis

The possible relationship between facial expression and depression can be traced back to the Darwin period (52). Strack et al. tested the hypothesis that people's facial activity affects their emotional responses (53). These results suggest that inhibition and promotion mechanisms may contribute to the observed emotional response (53). Other researchers have presented supportive results, indicating that signaling between the emotional center of the brain and the facial muscles is bidirectional, which further supports the facial feedback hypothesis (54).

It was confirmed that after the injection of BoNTs between the eyebrow and the orbicularis muscle, the depressed emotional state was reduced compared with depressed patients who had a saline injection. The improvement of depressed emotional state produced by BoNTs therapy was more obvious, when dealing with mild emotional stimuli (55). Wollmer et al. analyzed existing studies in an attempt to find better predictors to reflect the effects of BoNTs on patients with MDD. After data analysis, they found that high tension was the main predictor of BoNTs response, which sensitivity, specificity and overall accuracy of 100, 56, and 87%, respectively (56). The better effect of BoNTs for mood may be by the intervention of a proprioceptive feedback loop from the facial musculature to the emotional brain (57). Tension may be associated with more dynamic activities, resulting in more facial expression changes (58). In addition, BoNTs therapy reduced anxiety caused by frowning muscles, supporting the facial feedback hypothesis (59).

### Social Feedback Hypothesis

Through its action of reversible paralysis of mimic muscles, peripheral injection BoNT/A is considered to be able to prevent the emotional facial expression, including anger, sadness, and fear from being perceived by seeing of the face. Thus, BoNT/A therapy may improve the social interaction with people around, improve social contact, and then have benign social feedback. Namely, people would enter a virtuous circle of positive mood and social feedback, resulting in the persistent improvement of self-esteem. Furthermore, it was demonstrated that brain limbic system and mirror neuron system is involved in the recognition of emotional facial expression (60, 61).

### BDNF

The down-regulation of the expression of several neurotrophic factors has been involved in the pathogenesis of MDD. The most prominent and widespread representative is a polypeptide BDNF, which promotes cyclic adenosine monophosphate (cAMP) response element binding protein (CREB) phosphorylation through the activation of extracellular signal-regulated kinase (ERK). In the brain, especially in the hippocampus, BDNF plays a functional role in neuronal differentiation and survival, neurogenesis, synaptic plasticity, connectivity, maintenance of morphology, learning and memory (62). Intriguingly, BDNF levels in several brain regions are remarkably reduced in depression-like animals and depressed patients. Several commonly-used antidepressants, such as serotonin-selective reuptake inhibitors (SSRIs) (63) can up-regulate the level of BDNF expression in the hippocampus and the prefrontal cortex in order to exert antidepressant effects (64) (Figure 1). Our recent work also showed that facial injection of BoNT/A was also able to up-regulate the protein expression level of BDNF in the hippocampus not only at the mRNA but also at protein in mice. Furthermore, BoNT/A injection also activated the downstream ERK-CREB signaling pathways of BDNF in the hippocampus in stressed-mice (14). Thus, the up-regulation of BDNF levels may be a new mechanism underlying the therapeutic effects of BoNT/A in the management of depression.

### Monoamine Theory

The monoamine theory claims that there is an association between emotional disorders and reduced availability of the absence of 5-hydroxytryptamine (5-HT) or norepinephrine (NE) in the brain (65). Antidepressants can increase their brain content by inhibiting the reuptake of two important neurotransmitters (5-HT and NE) into nerve terminals (65). Some of the antidepressants based on monoamine theory include: tricyclic antidepressants (TCAs), monoamine oxidase inhibitors (MAOI), and SSRIs. Unilateral administration with BoNT/A into rat whisker significantly increased the NE levels in the striatum and 5-HT levels in the hypothalamus (14, 66). Interestingly, facial injection of BoNT/A significantly increased the 5-HT level in the hippocampus, hypothalamus, and prefrontal cortex in chronic stressed mice (14, 66). Thus, the results showed that BoNTs can up-regulate the levels of monoamines (e.g., 5-HT and NE) in the brain. Further clinical and basic studies need to identify the precise alteration of the neurotransmitters in the brain during BoNT/A therapy for depression.

### Insula cortex modification

Series of evidence support the importance of several brain areas associated with MDD, by using magnetic resonance imaging (MRI) (67–69) or meta-analysis the MRI data (70, 71). The most extensive research has been done on the insular cortex (IC), prefrontal cortex (PFC), anterior cingulate cortex (ACC), amygdala and hippocampus (62), which play a key role in sensation and emotion. IC is a brain region responsible for the coding of emotional and social aspects (51). Previous studies demonstrated abnormal insular connectivity may mediate mood disorders in the symptom of depression (72). A new hypothesis for the therapeutic effects of BoNTs on depression, refers to “insula cortex (IC) modification,” was recently proposed from transgenders' study (51). In patients suffering from mental illness, depression or bipolar disorder, it is possible to find both mental and physical dysphoria. The morphological changes of IC may be related to the difference between physical image and mental perception of one's body (51). Although the authors did not provide any evidence supporting BoNT/A therapy causes morphological alterations of IC, they proposed that the structural and functional modification of IC caused by BoNT/A therapy warrants further investigation.

### Neuroinflammation

An important part of the physiological stress-sensing system is the immune system, which interacts with main integrative systems of body, including the hypothalamic–pituitary–adrenal (HPA) axis, the autonomic nervous system and the central nervous system (CNS) (73). Immune disorders affecting CNS function would cause neuroinflammatory diseases, such as multiple sclerosis and autoimmune encephalitis. Low-level chronic systemic inflammation may play an important role in mediating the interface among psychological stress, depressive symptomatology, and association with depressive symptoms (74). However, there is no direct evidence to support the therapeutic mechanisms underlying the effects of BoNTs for depression involved in neuroinflammation. But there are some clues indicated the potential modulation effects of BoNTs on the development of neuroinflammation. The mechanisms underlying therapeutic effects of BoNTs on neuropathic pain are involved in restraining the release of inflammatory mediators and peripheral neurotransmitters from sensory nerves (75). Increased expression of inflammatory factors has been shown to be inhibited by monoclonal anti-Toll-like receptor 2 (TLR2) and inhibitors specific to intracellular proteins such as c-Jun N-terminal kinase (JNK), extracellular signal-regulated kinase (ERK), and p38 mitogen-activated protein kinase (MAPK) (76). Furthermore, BoNT/A treatment did not decrease LPS-induced release of pro-inflammatory factors in the astroglia, suggesting BoNT/A may have little effect on astrocytes (77). In contrast, BoNT/A treatment decreased the upregulation of expression of microglia-derived pro-inflammatory factors (77, 78), suggesting that BoNT/A may modulate the development of neuroinflammation by the inhibition of microglia activation in the CNS. The direct evidence supporting that therapeutic effects of BoNT/A on depression may owe to inhibiting neuroinflammation is still lacking.

## Future Perspective

Clinical and preclinical experiments have showed that BoNTs may be beneficial for the management of depression. However, the therapeutic application of BoNTs for depression has not USA or EU or Evidence-based therapeutic approved indication. The therapeutic mechanisms of BoNTs in the treatment for depression are needed to be further clarified. In view of the heterogeneity of the clinical findings and various influencing factors (16), more clinical trials should be performed to evaluate the dose equivalence, especially considering the different BoNT/A1 formulations. Notably, the potency of the preparations of BoNTs can be expressed as Units (U), where 1 U corresponds to 1 LD50 in the mouse bioassay (79). It is noteworthy that the clinical effect of 1 unit is not interchangeable between different preparations, because the bioassay methods used by different brands are different, (80).

Functional magnetic resonance imaging (fMRI) was used to assess neural responses to BoNTs treatment by radionuclide imaging in human (81). However, it remains a major challenge in objectively reflecting emotional state especially in animal models. Interestingly, a recent study showed that mice can display stereotyped facial expressions responding to emotionally salient events, and upon targeted manipulations in emotion-relevant neuronal circuits (82). By using brain imaging methods and monitoring facial expression of emotion in rodents, the therapeutic mechanisms of BoNT/A on depression may be better understood.

Recently, recombinant techniques (including site-directed mutations) have been used to create engineering botulinum neurotoxins as potential novel drugs for improving their therapeutic efficacy (83, 84). For instance, Yin et al. generated a “gain-of-function” mutation by replacing two non-aromatic residues at an extended loop in the C-terminal receptor-binding domain of BoNT/B (85). This engineering BoNT/B showed enhanced binding to neuronal membrane, enhanced efficacy in paralyzing muscles, and lowered systemic diffusion (85). In addition, BoNTs were designed as novel analgesic by utilizing the ability of BoNT to cleave SNARE complexes (83). Another engineering BoNT designed by mixing BoNT/E LC to the N-terminus of BoNT/A produced locally-applied and long-acting analgesic effects in a rat neuropathic pain model (86). Scheps et al. designed a mutant with three mutations (T420E; F423M; Y426F) in the C-terminus of BoNT/A1 light chain, which exert a faster onset and a shorter duration than wild-type BoNT/A1 (87). There are several lines of evidence supporting that BoNT/A1 therapy is generally safe and may be used as a new, alternative option for the treatment of depression (37). However, safety issue must be considered for all recombinant engineering BoNTs. Together, these novel engineering botulinum neurotoxins warrant further investigation for depression treatment.

## Conclusions

Although both clinical and preclinical studies have demonstrated that BoNT/A therapy may be an effective alternative intervention for depression, future investigations are needed to improve our understanding of the therapeutic mechanisms of BoNT/A for depression. Thus, studies on BoNT/A therapy may provide novel targets for the development of effective antidepressant drugs.

## Author Contributions

YL performed literature search and prepared the draft. TL and WL wrote the manuscript. All authors contributed to the article and approved the submitted version.

## Conflict of Interest

The authors declare that the research was conducted in the absence of any commercial or financial relationships that could be construed as a potential conflict of interest.
